# 'Tumour volume'.

**DOI:** 10.1038/bjc.1997.78

**Published:** 1997

**Authors:** C. A. Clelland


British Journal of Cancer (1997) 75(3), 464–465
© 1997 Cancer Research Campaign

Letters to Editor

### 'Tumour volume'

### Sir

We were interested to read the paper on survival after resection of non-small-cell lung cancer (NSCLC) in relation to 'tumour volume' by Jefferson et al (1996).

By multiplying together the three dimensions of each tumour, the authors have assumed that the carcinomas are box shaped. The accuracy of this assumption was not validated. In a study of 54 resected lung cancers (Binks et al, 1996), we found that the most appropriate measures of tumour volume were to assume that the tumours were ellipsoidal or boxes. These measurements compared well with our gold standard methods ($R$ = 0.887 and $R$ = 0.910 respectively) of sequential 1-mm or 1-cm slices, for which the tumour area was measured and the volume derived from the sum of the areas. In contrast, measurement of the maximum dimension and assuming a spherical shape grossly overestimated the volume of some tumours ($R$ = 0.632).

Although we expected ellipsoidal measurements to be more accurate than boxes, this was not the case. We support the authors statement that tumour volume is a useful piece of information that is easily collected, but three dimensions should always be measured for solid tumours.

Yours faithfully

*CA Clelland, Nottingham City Hospital, NHS Trust Hucknall Road, Nottingham, NG5 1PB UK*

### REFERENCES

Jefferson MF, Pendleton N, Faragher EB, Dixon GR, Myskow MW and Horan MA (1996) 'Tumour volume' as a predictor of survival after resection of non-small-cell lung cancer (NSCLC). *Br J Cancer* **74**: 456–459

Binks S, Clelland CA and Layton C (1996) A comparison of methods of measuring lung cancer volume. J Clin Pathol (in press).


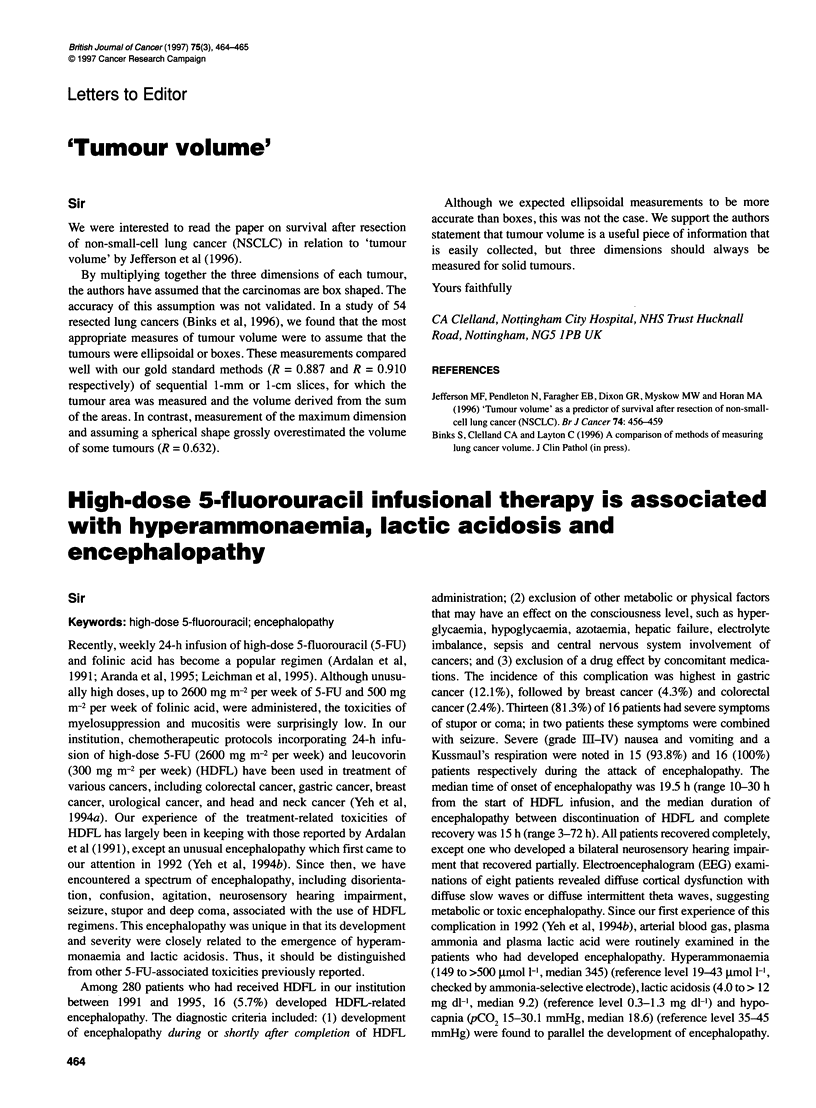


## References

[OCR_00040] Jefferson M. F., Pendleton N., Faragher E. B., Dixon G. R., Myskow M. W., Horan M. A. (1996). 'Tumour volume' as a predictor of survival after resection of non-small-cell lung cancer (NSCLC). Br J Cancer.

